# Systematic Review on the Applicability of Principal Component Analysis for the Study of Movement in the Older Adult Population

**DOI:** 10.3390/s23010205

**Published:** 2022-12-25

**Authors:** Juliana Moreira, Bruno Silva, Hugo Faria, Rubim Santos, Andreia S. P. Sousa

**Affiliations:** 1 Center for Rehabilitation Research–Human Movement System (Re)habilitation Area, Department of Physiotherapy, School of Health, Polytechnic of Porto, 4200-072 Porto, Portugal; 2 Research Center in Physical Activity, Health and Leisure, Faculty of Sports, University of Porto, 4200-450 Porto, Portugal; 3 School of Health, Polytechnic of Porto, 4200-072 Porto, Portugal; 4 Center for Rehabilitation Research–Human Movement System (Re)habilitation Area, Department of Physics, School of Health, Polytechnic of Porto, 4200-072 Porto, Portugal

**Keywords:** principal component analysis, biomechanics, kinematics, kinetics, older adults

## Abstract

Principal component analysis (PCA) is a dimensionality reduction method that has identified significant differences in older adults’ motion analysis previously not detected by the discrete exploration of biomechanical variables. This systematic review aims to synthesize the current evidence regarding PCA use in the study of movement in older adults (kinematics and kinetics), summarizing the tasks and biomechanical variables studied. From the search results, 1685 studies were retrieved, and 19 studies were included for review. Most of the included studies evaluated gait or quiet standing. The main variables considered included spatiotemporal parameters, range of motion, and ground reaction forces. A limited number of studies analyzed other tasks. Further research should focus on the PCA application in tasks other than gait to understand older adults’ movement characteristics that have not been identified by discrete analysis.

## 1. Introduction

The older adult population, those with age above 60 years, will increase in the coming decades in Europe [1]. Accounting for almost half of the total health costs [2], the aging population will increase the burden on healthcare services [3] expressed through an estimated increase from 3% to 4% in gross domestic product from 2004 to 2050 [4].

Biological aging can be defined as the progressive loss of function and represents a constant decrease in multisystemic capacity [5,6,7] that can be expressed in changes in movement patterns in various tasks [8]. Different biomechanical analyses have been associated with fall risk in older people, such as the displacement of the center of pressure in the standing position [9], decreased speed, stride length, and single limb support time in gait [10]. Other movement modifications were identified due to the aging process, such as decreased lumbar range of motion [11] during sit-to-stand, an indicator of functional independence in daily life [12]. However, because these findings have been obtained from discrete approaches, such as descriptive statistics and statistical inference based on only some parameters of the waveform [10,11], there is a consistent risk of information loss [13]. Additionally, other clinical measures assessed by classical psychometric procedures may have led to dubious conclusions [14], while others discarded several parts of the information and required a greater number of trials from participants before drawing conclusions [15]. Advanced multivariate analysis and machine learning methods have been applied to fully translate the complexity of the interactions between the variables [13]. Principal component analysis (PCA) is a multivariate statistical technique that reduces the volume of data to a smaller number, considering all the information captured [16,17]. PCA uses an orthogonal transformation to represent sets of potentially correlated variables with principal components (PC) that are linearly uncorrelated and are ranked so that the first PC has the largest possible variance. Accordingly, the PCs with the largest variance are selected to represent the correlated variables, resulting in a smaller volume of data, eliminating intercorrelations, and maintaining the ability to retain the majority of the information contained in the data initially obtained [16,18]. In the biomechanics context, PCA has become a useful method for analyzing a set of temporal waveforms of human motion data [17], and has been applied to the coordination of complex movement in different tasks of the upper [19] and lower limbs [20], population ages [21,22], and conditions [23,24,25]. Moreover, age-related changes in neuromuscular control, kinematics, and muscle function during gait identified by PCA were previously not detected by motion analysis through discrete variables [26]. Phinyomark et al., studying the biomechanical features of running gait data associated with iliotibial band syndrome, compared the results provided by discrete variables with the PCA. The authors suggest that care must be taken when selecting features of gait waveforms for both identification and discrimination of between-group differences for injured and non-injured runners [27]. Deluzio and Astephen first applied the PCA as a data reduction tool, as well as a preliminary step for further analysis to determine differences between the osteoarthritis and the control groups. The authors found that the discriminative features of the gait waveforms were the amplitude of the knee flexion moment, the range of motion of the knee flexion angle, the magnitude of the knee flexion moment during early stance, and the magnitude of the knee adduction moment during stance [28].

Currently, to the best of our knowledge, only one review addressed the degree of freedom and dimensional properties of gait task and the application of the PCA in a non-structured review [29]. However, it only addresses the gait task without specifying the population. Accordingly, this systematic review aims to synthesize current evidence regarding the study of the older adult’s movement (kinematics and kinetics) according to a PCA, compared to younger adults, summarizing the tasks and biomechanical variables studied. As a secondary aim, the instruments used to assess the tasks and biomechanical variables will be reported.

## 2. Materials and Methods

This systematic review was carried out according to the Preferred reporting items for systematic reviews and meta-analyses (PRISMA) statement 2020 [30], and was registered on the PROSPERO (International Prospective Register of Ongoing Systematic Reviews) platform with the registration number CRD42022329200 (https://www.crd.york.ac.uk/prospero/display_record.php?RecordID=329200, accessed on 23 October 2022).

### 2.1. Eligibility Criteria

According to the PICOS strategy, the criteria defined for inclusion of the studies were: population, older adults aged 60 years or over, living in the community; exposure, studies that applied the PCA in the biomechanical analysis of the movements of older adults; movement analysis associated with the aging process; comparator, young adults (less than 60 years); outcomes, tasks and biomechanical variables (kinematics and kinetics) and, if described, the instruments used to collect the biomechanical data; study design, observational studies (cross-sectional and longitudinal). As exclusion criteria, it was decided to exclude the studies if their participants were institutionalized (or if the data from institutionalized participants could not be separated from community-dwelling ones) or if the participants presented some major pathology with repercussions on the performance of movement, such as stroke or Parkinson disease (or if the data from participants with these conditions could not be separated from the complete study sample). The search was restricted to the studies published in the Portuguese and English languages, that were available in the last 20 years.

### 2.2. Selection and Data Collection Process

The studies were searched using three databases: MEDLINE (PubMed), Scopus and Web of Science (Supplementary Materials). Specific search algorithms were elaborated for each database, as described in the Table 1 for Pubmed and for the other two databases in the Supplementary Materials. Each concept was searched according to the database search instructions, using the MeSH terms and synonyms. Two reviewers independently assessed the studies’ titles and abstracts in the identification phase. Then, in the screening phase, the same reviewers assessed the full texts. Disagreements about whether a study should be included were resolved if there was an oversight of information on the part of one of the reviewers or by discussion, consulting a third reviewer in the cases of different interpretation of studies content. The two reviewers used a pre-defined table to extract data from the included studies.

### 2.3. Assessment of Methodologic Quality

The study design of the included studies is observational, and as there is no gold standard of risk of bias (ROB) tools for observational studies, different tools have been used in previous systematic reviews [31]. The most commonly used ROB tools are the Newcastle-Ottawa Scale (NOS) and the Downs & Black scale [31]. Downs & Black scale was found to be fairly comprehensive; easy to use and clear descriptions of how to score items [32]. The scale has good test-retest reliability (r = 0.88), good interobserver reliability (r = 0.75) and a high internal consistency (KR-20:0.89) [33]. Accordingly, it was decided to evaluate the ROB of articles included in this review by two reviewers. As in the previous processes, the differences between the two reviewers were solved by consulting a third reviewer. The Downs & Black instrument consists of 27 items that assess the quality of the study, including data reporting, external validity, internal validity (bias), internal validity (confounders) and power [33]. In this study, a modified version of the Downs & Black scale, adapted by Rollo et al., 2020, was used, in which the authors removed 10 items (8, 13–15, 17, 19, and 21–24) from the original scale, because they were considered not relevant for the analysis of observational studies. In addition, the authors modified the items (4, 5, 9, 26 and 27) and created two new items, one of which describes the criteria of internal validity and the other is related to the power of study [34]. The modified Downs & Black scale is then composed of 19 items, and the possible score on each item is 0 or 1. The maximum possible score is 19 points (all positive signs), with a higher score indicating higher quality [34].

Scores were given corresponding quality levels: excellent (18–19), good (14–17), fair (10–13), or poor (≤9) [34,35].

## 3. Results

**Figure 1:**
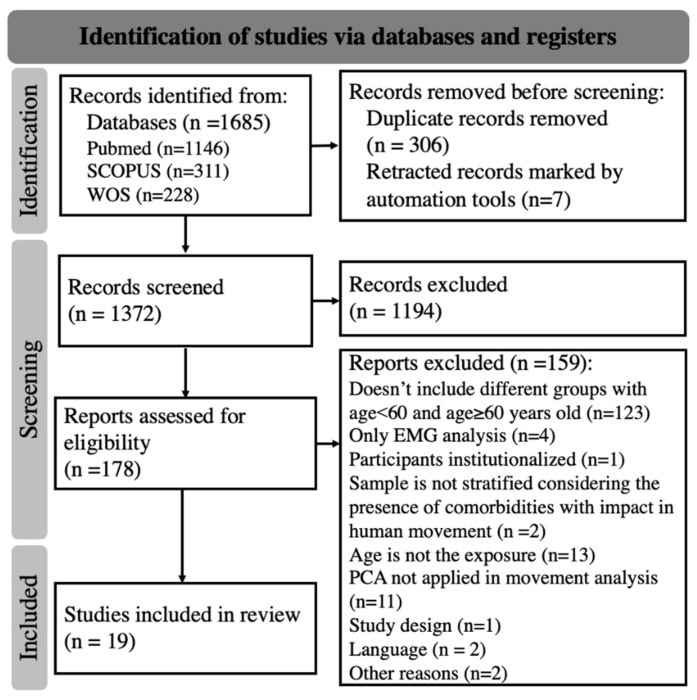
Systematic review PRISMA 2020 flow diagram.

The initial search was completed on 12 May 2022. From the 1685 results obtained in the data search, 306 duplicates were removed and seven studies were retracted by the automated tools. After the screening and posterior full-text analysis, 19 studies [36,37,38,39,40,41,42,43,44,45,46,47,48,49,50,51,52,53,54] were included in narrative analysis (Figure 1).

### 3.1. Characterization of the Included Studies

The identification, main purpose and conclusions of the 19 included studies is presented in Table 2. The publication year of each study varied between 2002 [50] and 2021 [37,49], and out of the nineteen studies, eighteen were cross-sectional, and one classified themselves as a retrospective cohort [39]. The study’s sample size ranged from 14 [46] to 239 [54] participants, constituting a total of 1281 participants. The older group sample size ranged from seven [46] to 127 [54] participants. The mean age of the older adults group ranged from 63, 7 years old [49] to 79, 43 years old [46], while the younger groups’ age ranged between 4.8 years [38] to 55,1 years old [54] (Table 3). Six studies did not present any information regarding the gender of the participants [36,40,41,44,47,53]. The mean percentage of female participants was 56.9% across the studies that reported this information. Moreover, there was one study that only included participants of the male gender [50], and one study that only included female participants [51].

Most of the included studies reported age-related differences between groups (*n* = 13) [37,39,40,41,42,44,45,46,47,48,51,53,54]. To understand if age had effects on the balance and coordination of movement, six studies were carried out [37,40,41,44,45,51]. Two included studies focused on the study of upper limbs [42,46]. Age-related differences were also reported by Boyer & Andriacchi, 2016 Reid et al., 2010, Rosenblum et al., 2020, Wu et al., 2007, and Zhou et al. 2020 when studying gait under different conditions. Also assessing gait under different conditions, five studies [36,38,49,50,52] found no age differences in intralimb coordination [36], segmental covariation [38], overall gait strategy [49], sagittal knee muscle moment curves [50], and in the regularity of whole-body movements [52].

### 3.2. Tasks Assessed in Included Studies

**Figure 2:**
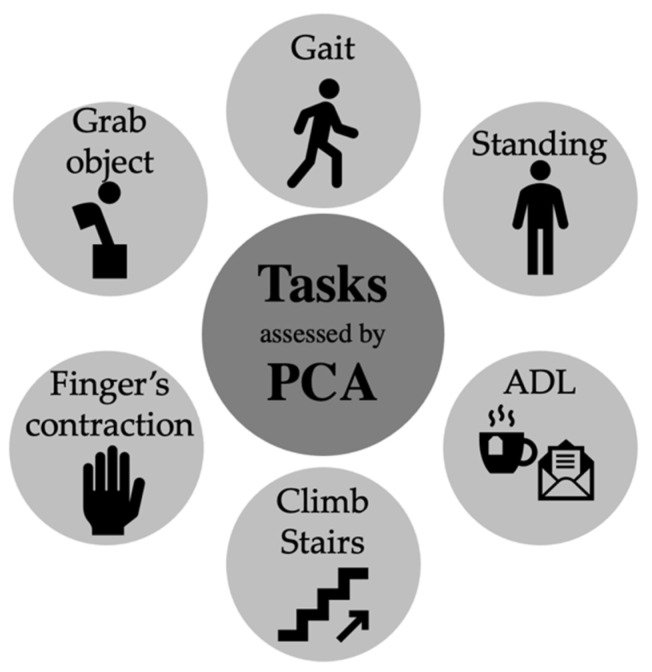
Summary of the older adults movement tasks analyzed and processed by PCA (ADL: Activity of daily living; PCA: Principal component analysis).

Eleven studies assessed the gait task. Five studies assessed the task in treadmill under perturbed conditions [36,48], different speeds [38,41], and dual tasking [52], while the other six studies assessed the overground gait [39,43,49,50,53,54]. Three studies assessed upright standing in unperturbed [44] and perturbed conditions [40,51]. The remaining five articles assessed different tasks, such as stepping [37], preparing a cup of tea and a letter [42], grasping an object placed at the ground from a standing position [45], climbing stairs [47], and during maximal voluntary contraction of fingers [46] (Figure 2).

### 3.3. Variables Assessed in Included Studies

**Figure 3:**
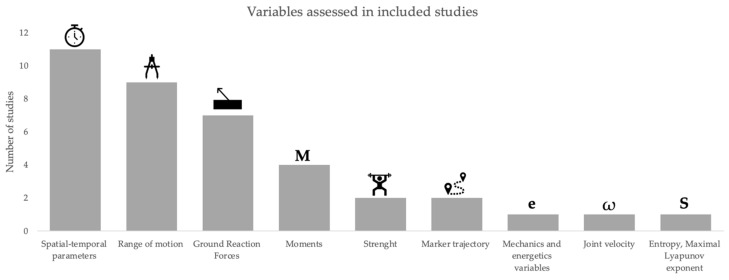
Summary of the older adults movement variables processed by PCA.

The spatial-temporal parameters [36,41,42,43,45,48,49,50,52,53,54], the range of motion (ROM) of lower limbs joints [36,38,39,40,41,43,47,49,51,53], and the ground reaction forces (GRF) [37,39,41,43,45,49,50] were the most assessed variables (Figure 3). Beyond these three variables the following variables have also been considered: calculation of moment [45,46,50], strength related variables [37,46,47], center of mass (CM) [40,51], center of pressure (CoP) [45,51], marker trajectory [37,52]; mechanics and energetics variables [38], joint velocity [44]; and multi scale entropy, cross-sample entropy, frequency variability, and maximal Lyapunov exponent [54].

### 3.4. Movement Analisys Instruments

For the movement capture and analysis, optoelectronic systems were used in all articles except two [46,54], where an inertial system [54] and force plates were used [46]. Among the seventeen studies that used optoelectronic systems, eight described the number of cameras used for capturing the movement [36,38,39,41,42,45,51,52]. The number of cameras used ranged from one [41] to twelve [52]. Force platforms were used in ten of the studies [37,39,41,43,45,47,48,49,50,51], in which six were studies that assessed gait [39,41,43,48,49,50] and the other four were related to stepping [37], stair climbing [47], and standing in the upright position [45,51].

### 3.5. The Use of PCA in Data Processing and/or Analysis

PCA was applied to extract features from several waveforms data [36,37,38,39,40,41,42,43,44,45,46,47,48,49,50,52]. The functional PCA was used in one of the included studies to produce a measure of variability across an entire curve captured as a small subset of functional principal components [51], and Kernel-based PCA was used in two studies, in one for nonlinear feature extraction and the evaluation of its effect on a subsequent classification in combination with learning algorithms (support vector machines) [53], and in the other for dimensionality data reduction for support vector machine classification [54].

### 3.6. Methodological Quality Assessment

The methodological quality assessment score, according to the Downs & Black scale, ranged from ten [36,40] to 16 [49] and is presented in Table 4. The average score of the articles included is approximately 12.79 points, the *fair* level. Seven studies [37,39,43,47,48,49,52] (36.8%) obtained a good classification and the remaining twelve (63.2%) obtained a fair classification. In general, the articles revealed very similar “Reporting” and “Internal validity” values, but only one scored in the “Power” section, while none scored in the “External validity” section.

## 4. Discussion

This systematic review aimed to summarize the tasks and biomechanical variables studied when PCA was applied in the study of the older adult population’s movement compared with younger adults. The results of the systematic search reinforce the need to gather this information as this method has been widely used, mainly in Europe (*n* = 7) and North America (*n* = 8), and more than half of the included studies (*n* = 11) were published in the last decade.

The study’s sample size ranged from 14 [46] to 239 [54] participants. In the literature, there is no agreement over the recommended sample size for the use of PCA, and the ratio between sample size and variables assessed. Guadagnoli and Velicer indicated that absolute minimum sample sizes, rather sample sizes as a function of the number of variables, are more relevant [55]. More recently, Osborne and Costello stated that both should be taken into consideration to avoid errors of interference, indicating that the best outcomes occur in analyses where large numbers of sample size and high ratios are present [56]. Because there is no simple method for calculating sample size in PCA [57], Comfrey and Lee (1992) cited by Osborne and Costello [56] suggested that “the adequacy of sample size might be evaluated very roughly on the following scale: 50–very poor; 100–poor; 200–fair; 300–good; 500–very good; 1000 or more–excellent”. In the present review, 11 of the 19 articles included have fewer than 50 participants, so interpretation of their results should take that into consideration.

The most assessed task was the gait, with five studies assessing the task on a treadmill [36,38,41,48,52], while the other six studies assessed the overground gait [39,43,49,50,53,54]. Older adults’ mobility can be influenced by multiple physiological and psychological factors [58], and other tasks as the balance should be assessed considering that its integrity is essential for activities of daily living efficacy [59]. Accordingly, only three studies assessed upright standing [40,44,51], all expressing promising results. Other activities, such as stair descent, which is regarded as one of the most difficult activities for older adults [60], are important to be assessed and processed in a broader context. In this review, only one study aimed to compare the gait patterns between young and older adults during stair climbing [47]. Therefore, there is a need to explore other tasks with the PCA approach. There are other activities, such as complex upper extremity-based manual activities of daily living tasks, in this review only assessed by Gulde et al. [42] which are still pending movement analyses based on kinematic markers [61], and consequently exploration by multivariate analysis.

Because gait was the most assessed task, the most assessed variables were related to the spatial-temporal parameters [36,41,42,43,45,48,49,50,52,53,54], and the ROM of lower limbs joints [36,38,39,40,41,43,47,49,51,53]. However, as stated by Richards, in gait, for example, the knee angular velocity has been shown to exhibit more sensitivity than the knee flexion angles and timing parameters alone [62]. As the joint angular velocity was only assessed by Liu et al. [44], it is necessary to explore different variables to better comprehend gait differences between older and younger adults.

PCA reduces the volume of data to a smaller number, and the visualization and statistical analysis of the new variables created, the principal components, can help to find similarities and differences between samples [16,63]. PCA was applied to extract features from several waveforms data [36,37,38,39,40,41,42,43,44,45,46,47,48,49,50,52]. In particular, PCA was used to analyze the angular covariance of the lower limb joints [38,40,41], extract space-time and kinematic data from gait [39,49], reduce the size of the data [37,47], and to assess motor coordination [36,42,44,45,52]. Additionally, the potential for PCA to uncover differences between groups was highlighted in three studies [43,48,50]. Accordingly, different applications of PCA were used within the included studies. Other studies used PCA variations, including functional PCA [51], and Kernel-based PCA [53,54]. Several included studies reported findings that were not possible by discrete analysis. Therefore, there is a need for the application of PCA in other tasks to understand older adult movement characteristics that have not been identified by discrete analysis. Cumbes and Azema proposed using the PCA to find feature patterns related to the autonomy–disability level, assessed by a disability scale, of elderly persons living in nursing homes [64]. In a longitudinal study, Shin et al. aimed to group diseases classified by the International Classification of Diseases using the PCA to extract comorbidity patterns and found that the principal component 1, which included diabetes, heart disease, and hypertension, was associated with an increased hazard ratio of mortality [65]. Some authors have already studied the kinematics of gait to cluster older adults with and without specific conditions [28,66,67]. The PCA clustering could be applied to kinematic and kinetic data of different daily performance tasks of community-dwelling older adults to cluster the autonomy–disability level and mortality. Early identification of those with disabilities and/or specific conditions could allow the introduction of prevention programs promoting older adults’ independence.

The results of this systematic review should be analyzed considering that three databases were searched. However, the three databases chosen include a broader range of indexed studies. Another limitation of this review may be the lack of inclusion of studies in languages other than English and Portuguese. A wider language criterion could increase the number of included studies as multivariate analysis has been used worldwide.

Taking into account that the vast majority of studies applied PCA to the analysis of tasks such as gait, as stated previously, it is necessary to develop studies that would investigate other tasks, including other daily life activities. In the upcoming studies, it is also necessary to include larger sample sizes in order to fully take advantage of the potential of multivariate analysis. Furthermore, other structured reviews and meta-analyses aiming to understand the role of PCA in the biomechanical analysis of older adults, differentiating between individuals with diseases or conditions and healthy ones [28,66,67], would be beneficial, as the evidence in these topics grows.

## 5. Conclusions

The aim of this systematic review was to gather the current information related to the use of the PCA method in the study of movement in the older adult population. Accordingly, PCA has been applied globally, mainly in the study of gait and orthostatic position. The main variables assessed were spatiotemporal parameters, the range of motion of lower limb joints, and ground reaction forces. PCA was mostly used to analyze the angular covariance of the lower limb joints, extract space-time and kinematic data from gait, reduce the size of the data, and assess motor coordination. A limited number of studies analyzed other tasks. Therefore, considering the potential of multivariate analysis, further research should focus on the PCA application in tasks other than gait to understand older adults’ movement characteristics that have not been identified by discrete analysis.

## Figures and Tables

**Table 1:** PICO framework strategy and search algorithm used in Medline (Pubmed) database. (* After a word, a truncation symbol (wildcard) to retrieve plurals or varying endings, # Truncation symbol for one character).

PICOS	Keyword	Algorithm	Query
Population	Older Adults	“aged”[MeSH Terms] OR “aged”[Title/Abstract] OR “elder *”[Title/Abstract] OR “older adult *”[Title/Abstract] OR “aged, 80 and over”[MeSH Terms] OR “aged 80 and over”[Title/Abstract] OR “older person *”[Title/Abstract] OR “centenarian *”[MeSH Terms] OR “centenarian *”[Title/Abstract] OR “sexagenarian *”[Title/Abstract] OR “septuagenarian *”[Title/Abstract] OR “octogenarian *”[MeSH Terms] OR “octogenarian *”[Title/Abstract] OR “nonagenarian *”[MeSH Terms] OR “nonagenarian *”[Title/Abstract]	#1
Exposure	PCA	“Principal Component Analysis”[MeSH Terms] OR “Principal Component Analysis”[Title/Abstract] OR “PCA”[Title/Abstract]	#2
Comparison	Young Adults	Did not restrict the comparator	
Outcomes	Tasks and biomechanical variables(kinematics and kinetics)	“Movement”[MeSH Terms] OR “Movement”[Title/Abstract] OR “Musculoskeletal Physiological Phenomena”[MeSH Terms] OR “Musculoskeletal Physiological Phenomena”[Title/Abstract] OR “Biomechanical Phenomena”[MeSH Terms] OR “Biomechanical Phenomena”[Title/Abstract] OR “movement evaluation”[Title/Abstract] OR “kinematics”[Title/Abstract] OR “biomechanics”[Title/Abstract] OR “Task Performance and Analysis”[MeSH Terms] OR “Task Performance and Analysis”[Title/Abstract] OR “task”[Title/Abstract] OR “gait”[MeSH Terms] OR “gait”[Title/Abstract] OR “sit-to-stand”[Title/Abstract] OR “Kinetics”[MeSH Terms] OR “kinetic *”[Title/Abstract] OR “Stair Climbing”[MeSH Terms] OR “Stair Climbing”[Title/Abstract] OR “Walking”[MeSH Terms] OR “Walking”[Title/Abstract] OR “Exercise Test”[MeSH Terms] OR “Exercise Test”[Title/Abstract]	#3
Study design	Observational studies	“randomized controlled trial”[Publication Type] OR “randomized controlled trials as topic”[MeSH Terms] OR “randomized controlled trial”[Title/Abstract] OR “clinical trial”[Publication Type] OR “clinical trial”[Title/Abstract] OR “controlled clinical trial”[Publication Type] OR “controlled clinical trials as topic”[MeSH Terms] OR “controlled clinical trial”[Title/Abstract] OR “Comment”[Publication Type] OR “Letter”[Publication Type] OR “correspondence as topic”[MeSH Terms] OR “Editorial”[Publication Type] OR “Review”[Publication Type] OR “review literature as topic”[MeSH Terms] OR “Systematic review”[Publication Type] OR “Systematic reviews as topic”[MeSH Terms] OR “Systematic review”[Title/Abstract] OR “meta analysis”[Publication Type] OR “meta analysis as topic”[MeSH Terms] OR “meta analysis”[Title/Abstract] OR “meta analysis as topic”[MeSH Terms] OR “Guideline”[Publication Type] OR “Practice Guideline”[Publication Type] OR “Practice Guidelines as Topic”[MeSH Terms]	#4
Final Query		(#1 AND #2 AND #3) NOT #4	

**Table 2:** The identification, main purpose and conclusions of the included studies.

Study	Purpose	Conclusion
Aprigliano, et al. 2017 [36] Italy	Assess how aging modifies intralimb coordination strategy during corrective responses during treadmill walking.	Intralimb coordination described by the planar covariation law was not affected by aging.
Armstrong, et al. 2021 [37] Canada	Assess if the whole-body movement and/or motor control strategy differ as a function of age or sex in a forward reactive step to maintain balance.	PCA enabled to differentiate younger and older adults according to gender in terms of whole-body reactive stepping strategy and how ground reaction forces and kinetics support maintaining balance synergistically with whole-body movement strategy, when combined with multiple regression analysis.
Bleyenheuft & Detrembleur, 2012 [38] Belgium	Assess the impact of age on kinematic segmental covariation at 3 different walking speeds.	The covariation remains stable between 15 and 70 years old.
Boyer & Andriacchi, 2016 [39] United States of America (USA)	Assess the impact of age on knee function during walking in individuals with healthy knees as it applies to the development of knee osteoarthritis.	PCA analysis provided insight to the progressive changes in the magnitude of joint angles and in the kinematic coupling at the knee with age.
De Freitas et al., 2010 [40] Brazil	Assess age-related effects on postural responses following forward support surface translation throughout middle-adulthood and early old age.	Independent of age, the individuals were able to minimize center of mass backward displacements in response to forward perturbation and to revert the direction of this displacement at proper time with similar kinematics patterns. However, after the fifth decade changes in neuromuscular responses are observed.
Dewolf et al., 2019 [41] Belgium	Assess the effects of age on the intersegmental coordination in healthy young and elderly adults walking at matched speeds.	Older adults present decreased intersegment covariation with speed compared to young adults, mainly related to foot-shank coordination.
Gulde et al., 2019 [42] Germany	Assess the effects of speed of execution on upper-limb kinematics, in activities of daily living, with respect to age.	PCA revealed a movement strategy and age-dependent decline in primarily executive functions.
Kobayashi et al., 2016 [43] Japan	Assess age independent and most dominant sex differences observed in gait during normal walking.	PCA was able to identify a variation with significant age-sex interaction and another with significant sex difference but no age-effect or age-sex interaction.
Liu et al., 2020 [44] Taiwan	Assess the coordination of the multiple joints of the human body to maintain a stable posture and how it varies with age.	Aging increases the coupling strength and decreases the changing speed and the complexity of inter-joint coordination patterns.
Paizis et al., 2008 [45] France	To understand equilibrium function and movement coordination in elderly by means of a whole-body goal-oriented task.	During whole-body movements, center of mass displacements are smaller in elderly compared to young adults and this postural aging effect is associated with straighter wrist paths. Despite these changes, high covariations of joint and elevation angles, observed in young adults, were also preserved in older adults.
Park et al., 2011 [46] USA	Assess age-related changes in finger coordination during accurate force and moment of force production tasks	The magnitudes of the loading coefficients in the PC analysis suggested that the young subjects used mechanical advantage to produce moment while elderly subjects did not.
Reid et al., 2010 [47] Canada	To use PCA to compare the gait patterns between young and older adults during stair climbing	The PCA and discriminant function analysis identified gait pattern differences between young and older adults.
Rosenblum et al., 2020 [48] Israel	To calculate total recovery time after different types of perturbations during walking and use it to compare young and older adults following different types of perturbations.	PCA showed differences in step length and step width recovery times between AP and ML perturbations.
Rowe et al., 2021 [49] Canada	To examine and describe age and sex-specific temporal pattern differences in lower extremity gait mechanics in asymptomatic adults.	The use of PCA enabled the observation of major sex-specific differences leading to the identification of an overall difference in walking gait strategy between healthy adult male and female participants, independent of age.
Sadeghi et al., 2002 [50] Canada	To identify the main structural characteristics of the sagittal knee muscle moment curves developed in elderly and young able-bodied subjects	No significant differences were found between groups about the quality or magnitude of the sagittal knee peak muscle moment during the stance phase and early swing phase
Slaboda, J. C., 2011 [51] USA	To explore the influence of continuous visual flow, during and following a postural disturbance (i.e., support surface tilt), on the ability to reorient to vertical.	The fPCA revealed greatest mathematical differences in center of mass and center of pressure responses between groups or conditions during the period that the platform transitioned from the sustained tilt to a return to neutral position
Verrel et al., 2009 [52] Germany	To investigate the effects of concurrent cognitive task difficulty (n-back) on the regularity of whole-body movements during treadmill walking in women and men from 3 age groups.	Age seems to not influence gait regularity.
Wu et al., 2007 [53] China	To evaluate the use of Kernel-based Principal Component Analysis to extract more gait features (i.e., to obtain more significant amounts of information about human movement) and improve the classification of gait patterns.	Nonlinear gait features can be extracted to automatic classification of healthy young or older adults gait patterns.
Zhou et al., 2020 [54] Netherlands	To evaluate if different groups (healthy young-middle aged adults, healthy older adults, and geriatric patients) can be classified based on dynamic outcomes.	The following dynamic gait outcomes are important for classifying the three groups: regularity (vertical direction), stability (maximal Lyapunov exponent of the vertical acceleration) and pace (gait speed and the variability of the accelerations (RMS) in anterior-posterior and vertical direction).

**Table 3:** Included studies’ characterization, specifically, the study identification, sample size, mean age, and percentage of females, the tasks, variables, instruments and description of the PCA use in the study.

Study	Participants (*n*) Females (%) Age (Mean ± SD)	Tasks	Variables	Instruments	PCA in Data Processing and/or Analysis
Aprigliano, et al. 2017 [36]	20 10 YG 24.4 ± 2.5 10 OG 66.3 ± 5.1	Treadmill gait with and without perturbations	Spatio-temporal parameters stride duration; stance phase duration; step length; step width Range of Motion (ROM) Hip; Knee; Ankle Intralimb coordination	Optoelectronic system -Vicon Motion Analysis System (Oxford, UK) -6 cameras	PCA was used to assess intralimb coordination calculated through the relationship among elevation angles (planar covariation law).
Armstrong, et al. 2021 [37]	80, 56, 25% 40 YG M 23.0 ± 2.8; F 22.3 ± 3.7 40 OG M 71.6 ± 3.7 F 68.3 ± 4.2	Hip and knee maximal isometric contraction; Stepping.	Strength: -Hip flexion, extension and abduction -Knee extension; Marker trajectory; Voluntary reaction time; Ground reaction forces (GRF)	Uni-axial load cell -MLP-300-CO, Transducer Techniques, Temecula, CA. Optoelectronic system: -Optotrak Certus, NDI (Waterloo, ON, Canada). Force platform: OR6-7, Advanced Mechanical Technology Incorporated, USA	PCA was used to reduce the dimensionality of time-series, marker trajectory data captured to represent whole-body stepping responses.
Bleyenheuft & Detrembleur, 2012 [38]	30 6 5 y: 4.8 ± 0.4, 83% 6 10 y 9.3 ± 0.5, 50%F 6 15 14.3 ± 0.5, 100% 6 20 y 23.5 ± 2.9, 100% 6 70 y 77.3 ± 5.0, 50%	Treadmill gait at 3 different speeds: 1 km h−1, 3 km h−1, and 5 km h−1,	ROM: -thigh, shank and foot elevation angles; Mechanics and energetics Mechanical power Energetic cost	Optoelectronic system: -ELITE system -6 cameras Ergospirometer (Cosmed, Rome, Italy)	PCA was used to describe the covariation between thigh, shank and foot elevation angles.
Boyer & Andriacchi, 2016 [39]	74 25 YG 24 ± 2.3, 44% 25 MAG 48 ± 4.7, 48% 24 OG 64 ± 2.4, 54%	Overground gait at self-selected speed	ROM: -knee flexion, ab-adduction and internal-external rotation angles; Anterior-posterior translation of the tibia with respect to the femur forces GRF	Optoelectronic system: -ProReflex, Qualysis Inc, Sweden -8 cameras Force platform: -Bertec Corporation, Columbus, OH, USA	PCA was used to characterize and statistically compare the patterns of joint movement and identifying interactions between the three components of joint rotation and the translation.
de Freitas et al., 2010 [40]	36 9 20–25 y 9 40–45 y 9 50–55 y 9 60–65 y	stand on the platform to evaluate the participants’ postural reactions to temporally unpredictable perturbations	ROM: -ankle, knee, and hip Maximum backward displacement of body center of mass (CM) time-to-reversal of body CM	Optoelectronic system: -Optotrak (Digital Northern, Inc.).	PCA was performed on the linear covariation of ankle, knee, and hip joint angles to estimate the postural synergies [i.e., the coupling among the joints involved in the postural task] employed to minimize the CM horizontal displacement
Dewolf et al., 2019 [41]	26 8 YG 24.5 ± 2.4 18 OG 75.6 ± 6.7	treadmill gait at 6 different selected speeds (0.56, 0.83, 1.11, 1.39, 1.67 and 1.94 m s−1)	GRF ROM: -hip, knee, and ankle) Spatiotemporal parameters: -Stride length	Modified commercial treadmill -h/p/ComosStellar, Germany -4 force transducers (Arsalis, Belgium). Optoelectronic system: high-speed video camera (BASLER piA 640-210).	PCA was applied to determine the covariance matrix of the hip, knee and ankle elevation angles
Gulde et al., 2019 [42]	64 26 YG 22.31 ± 2.13, 58% 16 OG 63.06 ± 2.43, 50% 22 RG 71.27 ± 3.48, 50%	To prepare a cup of instant ice-tea or to prepare a letter to be sent and performed at a natural speed or as fast as possible.	Spatiotemporal parameters: trial duration, relative activity, path length, relative vertical path length, mean peak velocity, number of velocity peaks per meter, bimanual cooperation, and quotient, bimanual velocity ratio	Optoelectronic system: -Qualisys Inc., Gothenburg, Sweden -7 cameras	PCA was used to extract the underlying relationship between age, activities of daily living performance, executive functions (trail making tasks), and fine motor control (Nine-Hole Peg Tests)
Kobayashi et al., 2016 [43]	191 67 YG 27.21 ± 5.37, 54% 43 MAG 52.74 ± 7.55, 49% 81 OG 68.01 ± 2.82, 43%	overground gait at comfortable, self-selected speed	ROM pelvic, right hip, knee, and ankle Spatiotemporal parameters walking speed, step length, step width, stance time, swing time GRF	Optoelectronic system: -3D motion capture system (VICON) Force platform: -Force plates (BP400600-2000PT, AMTI)	PCA was used to identify the most dominant age independent sex differences in gaits during normal gait
Liu et al., 2020 [44]	45 15 YG 24.06 ± 2.02 30 OG 71.13 ± 4.56	standing still in a comfortable stance for 40 s.	Joint velocity signals: Mediolateral signals of the joints’ center	Optoelectronic system: - Microsoft Kinect V2 sensor - Five-point stencil	PCA was performed on the joint velocity vectors for each experimental trial to quantify the complexity of inter-joint coordination pattern from a global perspective
Paizis et al., 2008 [45]	16 8 YG 23 ± 1.51, 50% 8 OG 74.5 ± 4.5, 50%	From standing posture, participants were requested to make a whole-body movement in the sagittal plane to grasp a wooden dowel placed at ground level in front of them.	Spatiotemporal parameters: -movement duration, peak velocity, path displacement, path deviation from straightness, path curvature Position of the center of pressure (CoP) Amplitude of the CoP displacement and backward direction GRF	Optoelectronic system: -SMART (BTS, Milan) -5 cameras Force Platform: AMTI (Advanced Mechanical Technology Inc., Watertown, MA)	PCA was performed to evaluate the whole-body movement coordination. To account for different motor strategies separate PCA were performed for each participant and for each condition
Park et al., 2011 [46]	14 7 YG 29.86 ± 2.27, 71% 7 OG: 79.43 ± 4.31, 43%	Maximal voluntary contraction tasks and accurate force–moment production tasks, performed by the index finger and by four fingers pressing together	Strength: total normal force (FTOT) and moment of normal force (MTOT)	Force sensors: -Nano-17, ATI Industrial Automation, Garner, NC	PCA was performed on the finger force data which covered a broad range of FTTOT and MTOT combinations
Reid et al., 2010 [47]	62 30 YG 23.9 ± 2.6 32 OG 65.5 ± 5.2	Stair climbing	ROM Knee Flexion, Internal rotation, Adduction Posterior–anterior, Lateral–medial, Distal–proximal force Flexion, Internal rotation moment net forces and net reaction moments at the knee	Optoelectronic system: Optotrak 3020 (Northern, Digital, Waterloo, Canada) Force platform: Force plate (AMTI, Newton, MA, USA)	PCA was used to reduce the size of the data set. PCs were created for the knee joint moment, angle, and force curves about the three axes
Rosenblum et al., 2020 [48]	24 12 YG 26.92 ± 3.40, 71% 12 OG 66.83 ± 1.60, 50%	treadmill gait with medio-lateral (ML) or anterior-posterior (AP) perturbations	Spatiotemporal parameters -step length, step width, total recovery time	Optoelectronic system: -Motek-Medical, the Netherlands Force plates: Zemic load cells; The Netherlands	PCA was used to explore the effects of perturbation direction on total recovery times, applying the singular value decomposition
Rowe et al., 2021 [49]	154 38 20-40y: 34.7 ± 5.9, 66% 45 41-50y: 46.2 ± 2.7, 67% 47 51-59y 55.1 ± 2.6, 64% 24 60 + Y 63.7 ± 3.5, 38%	overground gait in self-selected speed	ROM ankle, knee and hip) Spatiotemporal parameters walking speed, stride length, stance time GRF	Optoelectronic system: Optotrak motion capture system (Northern Digital, Inc.) Force platform: force platform (AMTI, Watertown, MA, USA).	PCA was applied to extract major patterns of variability from hip, knee and ankle joint angles and net external moments
Sadeghi et al., 2002 [50]	40, 0% 20 YG 25 ± 8.1 20 OG 72 ± 5.5	overground gait at self-selected pace	Spatiotemporal parameters -speed, stance phase, stride length, cadence GRF	Optoelectronic System Motion Analysis system (YG) Optotrak (OG) Force plates AMTI	PCA was applied as a classification and data structure detection method to the sagittal knee muscle moment curves of the elderly and young subjects
Slaboda, J. C., 2011 [51]	28, 100% 14 YG (20–39) 14 OG (60–79)	Standing in the upright position in the dark while different tilts were applied to the platform	COP CM ROM ankle and hip	Force platform -AMTI, Watertown, MA. Optoelectronic System Motion Analysis (Santa Rosa, CA, USA) 6 cameras	Functional PCA was applied to CM, COP, segmental angles, and IMNF (instantaneous mean frequency curve) data to identify trial periods in which the two populations were differentially affected by visual conditions
Verrel et al., 2009 [52]	96 32 20–30y, 50% 32 60–70y, 50% 32 70–80y, 50%	overground gait at a fixed speed (2.5 km/hr.) and self-selected speed while dual tasking	Spatiotemporal parameters: -Stride frequency, Step cycle Marker trajectory	Optoelectronic System: motion (Vicon 612, Workstation 4.6; Vicon Ltd., Oxford, UK) 12 cameras (infrared V-cam 100 & 200)	The PCA was used to assess gait regularity based on the split of marker trajectory into residual and main components
Wu et al., 2007 [53]	48 24 YG 25.10 ± 5.3 24 OG 74.6 ± 2.55	overground gait at a self-selected speed	Spatiotemporal parameters Stride length, Stride duration, Gait velocity, Single support duration, stance duration, Swing duration, Gait cadence ROM: Hip, knee and ankle	Optoelectronic System: OptoTrak 3020 motion analysis system (Northern Digital Inc., Waterloo, Canada).	The PCA and KPCA were used to extract nonlinear features from spatiotemporal and kinematic gait data for automatic classification of healthy young or older adults gait patterns
Zhou et al., 2020 [54]	239 57 MAG 42.72 ± 16.6, 47% 55 OG 74.58 ± 5.71, 36% 127 RG 79.3 ± 5.81 51%	overground gait	Spatiotemporal parameters: -Speed, gait step or stride regularity, Root Mean Square, Smoothness: Index of Harmonicity, symmetry, multiscale Entropy, Cross-sample Entropy, frequency variability, maximal Lyapunov exponent	iPod Touch G4 (iOS 6; Apple Inc.) accelerometer unit: DynaPort hybrid unit (McRoberts BV, Te Hague, The Netherlands)	The PCA and KPCA were used to reduce the dimensionality of calculated outcomes while preserving the informative and variability properties

CM, centre of mass; CoP, centre of pressure; F, female; Total normal force, FTOT; Moment of normal force, MTOT; GRF, ground reaction force; M, male; MAG, middle aged adults; OG, older group; RG, retiree/geriatric group; ROM, range of motion; SD, standard deviation; y, years; YG, young group.

**Table 4:** Methodological quality assessment according to modified Downs & Black scale.

	Modified Downs & Black Scale Items																			
Study ID	1	2	3	4	5	6	7	8	9	10	11	12	13	14	15	16	17	18	19	Total
Aprigliano, et al., 2017 [36]	1	1	0	0	0	1	1	1	0	0	0	1	0	1	1	1	1	0	0	10
Armstrong, et al., 2021 [37]	1	1	1	0	1	1	1	1	1	0	0	1	1	1	1	1	1	0	0	14
Bleyenheuft & Detrembleur, 2012 [38]	1	1	0	0	0	1	1	1	1	0	0	1	1	1	1	0	1	0	0	11
Boyer & Andriacchi., 2016 [39]	1	1	1	1	1	1	1	1	1	0	0	1	1	1	1	1	1	0	0	15
de Freitas et al., 2010 [40]	1	1	0	0	0	1	1	1	0	0	0	1	1	1	1	0	1	0	0	10
Dewolf et al., 2019 [41]	1	1	0	0	0	1	1	1	1	0	0	1	1	1	1	0	1	0	0	11
Gulde et al., 2019 [42]	1	1	1	0	1	1	1	1	1	0	0	1	1	1	1	1	1	0	0	13
Kobayashi et al., 2016 [43]	1	1	1	0	1	1	1	1	1	0	0	1	1	1	1	1	1	0	0	14
Liu et al., 2020 [44]	1	1	0	0	1	1	1	1	1	0	0	1	1	1	1	1	1	0	0	13
Paizis et al., 2008 [45]	1	1	0	0	1	1	1	1	0	0	0	1	1	1	1	1	1	0	0	12
Park et al., 2011 [46]	1	1	1	0	0	1	1	1	0	0	0	1	1	1	1	0	1	0	0	11
Reid et al., 2010 [47]	1	1	1	0	1	1	1	1	1	0	0	1	1	1	1	1	1	0	0	14
Rosenblum et al., 2020 [48]	1	1	1	0	1	1	1	1	1	0	0	1	1	1	1	1	1	0	0	14
Rowe et al., 2021 [49]	1	1	1	0	1	1	1	1	1	0	0	1	1	1	1	1	1	1	1	16
Sadeghi et al., 2002 [50]	1	1	1	0	1	1	1	1	0	0	0	1	1	1	1	1	1	0	0	13
Slaboda, J. C., 2011 [51]	1	1	1	0	1	1	1	1	0	0	0	1	1	1	1	1	1	0	0	13
Verrel et al., 2009 [52]	1	1	1	0	1	1	1	1	1	0	0	1	1	1	1	1	1	0	0	14
Wu et al., 2007 [53]	1	1	1	0	1	1	1	1	UD	0	0	1	1	1	1	1	1	0	0	13
Zhou et al., 2020 [54]	1	1	1	0	1	1	1	1	0	0	0	1	0	1	1	1	1	0	0	12

## Data Availability

Not applicable.

## Supplementary Materials

The following supporting information can be downloaded at: https://www.mdpi.com/article/10.3390/s23010205/s1. Figure S1: Specific search algorithm for Scopus; Figure S2: Specific search algorithm for Web of Science; Figure S3: Specific search algorithm for Pubmed.
