# Supplementary material for: Systematic Review on the Applicability of Principal Component Analysis for the Study of Movement in the Older Adult Population
Source: Sensors (Basel). 2022 Dec 25;23(1):205. doi: 10.3390/s23010205 (PMC9823400; doi:10.3390/s23010205)
Supplement: Supplementary file 1 [file sensors-23-00205-s001.zip › sensors-2019705-supplementary.pdf]

Supplementary material

Systematic Review on the Applicability of Principal Component Analysis for the Study of Movement in the Older Adult Population

Juliana Moreira [1,2], Bruno Silva [3], Hugo Faria [3], Rubim Santos [4] and Andreia S. P. Sousa [1,*]

Database search - Scopus

Search history — Combine queries... *e.g. #1 AND NOT #3*

18	((TITLE-ABS-KEY ("aged" OR "elder*" OR "older adult*" OR "aged, 80 and over" OR "older person*" OR "centenarian" OR "sexagenarian*" OR "septuagenarian*" OR "octogenarian*" OR "nonagenarian*")) AND (TITLE-ABS-KEY ("Movement" OR "Musculoskeletal Physiological Phenomena" OR "Biomechanical Phenomena" "biomechanics" OR "movement evaluation" OR "Task Performance and Analysis" OR "task" OR "gait" OR "sit-to-stand" OR "Stair Climbing" OR "Walking" OR "kinetic*" OR "Exercise Test")) AND (TITLE-ABS-KEY··· View More	311 document results
17	((TITLE-ABS-KEY ("aged" OR "elder*" OR "older adult*" OR "aged, 80 and over" OR "older person*" OR "centenarian" OR "sexagenarian*" OR "septuagenarian*" OR "octogenarian*" OR "nonagenarian*")) AND (TITLE-ABS-KEY ("Movement" OR "Musculoskeletal Physiological Phenomena" OR "Biomechanical Phenomena" "biomechanics" OR "movement evaluation" OR "Task Performance and Analysis" OR "task" OR "gait" OR "sit-to-stand" OR "Stair Climbing" OR "Walking" OR "kinetic*" OR "Exercise Test")) AND (TITLE-ABS-KEY··· View More	322 document results
16	(TITLE-ABS-KEY ("randomized controlled trial" OR "controlled clinical trial" OR "clinical trial" OR "systematic review" OR "meta-analysis"))	2,398,186 document results
15	(TITLE-ABS-KEY ("aged" OR "elder*" OR "older adult*" OR "aged, 80 and over" OR "older person*" OR "centenarian" OR "sexagenarian*" OR "septuagenarian*" OR "octogenarian*" OR "nonagenarian*")) AND (TITLE-ABS-KEY ("Movement" OR "Musculoskeletal Physiological Phenomena" OR "Biomechanical Phenomena" "biomechanics" OR "movement evaluation" OR "Task Performance and Analysis" OR "task" OR "gait" OR "sit-to-stand" OR "Stair Climbing" OR "Walking" OR "kinetic*" OR "Exercise Test")) AND (TITLE-ABS-KEY··· View More	359 document results
14	TITLE-ABS-KEY ("Principal Component Analysis" OR "PCA")	255,172 document results
13	TITLE-ABS-KEY ("Movement" OR "Musculoskeletal Physiological Phenomena" OR "Biomechanical Phenomena" "biomechanics" OR "movement evaluation" OR "Task Performance and Analysis" OR "task" OR "gait" OR "sit-to-stand" OR "Stair Climbing" OR "Walking" OR "kinetic*" OR "Exercise Test")	206,049 document results
12	TITLE-ABS-KEY ("aged" OR "elder*" OR "older adult*" OR "aged, 80 and over" OR "older person*" OR "centenarian" OR "sexagenarian*" OR "septuagenarian*" OR "octogenarian*" OR "nonagenarian*")	6,010,627 document results

Showing all recent searches | View 5 most recent only

Top of page

Figure S1: Specific search algorithm for Scopus

Database search - Web of Science

#	Query	Results
7	**#4 NOT #5** and **2022** or **2021** or **2020** or **2019** or **2018** or **2017** or **2016** or **2015** or **2014** or **2013** or **2012** or **2011** or **2010** or **2009** or **2008** or **2007** or **2006** or **2005** or **2004** or **2003** or **2002** (Publication Years)	228
6	**#4 NOT #5**	245
5	**TS=(("randomized controlled trial" OR "controlled clinical trial" OR "clinical trial" OR "systematic review" OR "meta-analysis"))**	758,491
4	**#1 AND #2 AND #3**	254
3	**TS=((“Principal Component Analysis" OR "PCA”))**	166,672
2	**TS=((“Movement" OR “Musculoskeletal Physiological Phenomena” OR “Biomechanical Phenomena” "biomechanics” OR “movement evaluation” OR “Task Performance and Analysis” OR “task” OR “gait” OR ”sit-to-stand” OR “Stair Climbing” OR “Walking” OR “kinetic*” OR “Exercise Test”))**	2,725,781
1	**TS=((“aged" OR "elder*” OR “older adult” OR “older person*” OR “aged, 80 and over” OR “centenarian*” OR “sexagenarian*” OR “septuagenarian*” OR “octogenarian*” OR “nonagenarian*”).)**	1,007,230

Figure S2: Specific search algorithm for Web of Science

Database search - PubMed

Search	Actions	Details	Query	Results	Time
#11	···	>	Search: **#4 NOT #5** Filters: **from 2002 - 2022**	1,146	06:32:14
#7	···	>	Search: **#4 NOT #5** Filters: **from 2000 - 2022**	1,166	05:53:50
#10	···	>	Search: **#9 AND #2 AND #3**	37	05:51:18
#9	···	>	Search: **#1 AND #8**	31,824	05:50:52
#8	···	>	Search: **("independent living"[MeSH Terms] OR "independent living"[Title/Abstract] OR "community dwelling"[Title/Abstract] OR "community-dwellers"[Title/Abstract] OR "community living"[Title/Abstract] OR "aging in place"[Title/Abstract])**	37,458	05:50:30
#6	···	>	Search: **#4 NOT #5**	1,266	05:37:30
#5	···	>	Search: **("randomized controlled trial"[Publication Type] OR "randomized controlled trials as topic"[MeSH Terms] OR "randomized controlled trial"[Title/Abstract] OR "clinical trial"[Publication Type] OR "clinical trial"[Title/Abstract] OR "controlled clinical trial"[Publication Type] OR "controlled clinical trials as topic"[MeSH Terms] OR "controlled clinical trial"[Title/Abstract] OR "Comment"[Publication Type] OR "Letter"[Publication Type] OR "correspondence as topic"[MeSH Terms] OR "Editorial"[Publication Type] OR "Review"[Publication Type] OR "review literature as topic"[MeSH Terms] OR "Systematic review"[Publication Type] OR "Systematic reviews as topic"[MeSH Terms] OR "systematic review"[Title/Abstract] OR "Meta-Analysis"[Publication Type] OR "Meta analysis as Topic"[MeSH Terms] OR "Meta analysis"[Title/Abstract] OR "Meta-analysis as Topic"[MeSH Terms] OR "Guideline"[Publication Type] OR "Practice Guideline"[Publication Type] OR "Practice Guidelines as Topic"[MeSH Terms])**	6,344,627	05:36:37
#4	···	>	Search: **#1 AND #2 AND #3**	1,502	05:33:58
#3	···	>	Search: **("Principal Component Analysis"[MeSH Terms] OR "Principal Component Analysis"[Title/Abstract] OR "PCA"[Title/Abstract])**	92,033	05:33:22
#2	···	>	Search: **("Movement"[MeSH Terms] OR "Movement"[Title/Abstract] OR "Musculoskeletal Physiological Phenomena"[MeSH terms] OR "Musculoskeletal Physiological Phenomena"[Title/Abstract] OR "Biomechanical Phenomena"[MeSH Terms] OR "Biomechanical Phenomena"[Title/Abstract] OR "movement evaluation"[Title/Abstract] OR "kinematics"[Title/Abstract] OR "biomechanics"[Title/Abstract] OR "Task Performance and Analysis"[MeSH Terms] OR "Task Performance and Analysis"[Title/Abstract] OR "task"[Title/Abstract] OR "gait" [MeSH Terms] OR "gait"[Title/Abstract] OR "sit-to-stand"[Title/Abstract] OR "Kinetics"[MeSH Terms] OR "kinetic*"[Title/Abstract] OR "Stair Climbing"[MeSH Terms] OR "Stair Climbing"[Title/Abstract] OR "Walking"[MeSH Terms] OR "Walking"[Title/Abstract] OR "Exercise Test"[MeSH Terms] OR "Exercise Test"[Title/Abstract])**	2,598,220	05:31:42
#1	···	>	Search: **("aged"[MeSH Terms] OR "aged"[Title/Abstract] OR "elder*"[Title/Abstract] OR "older adult*"[Title/Abstract] OR "aged, 80 and over"[MeSH Terms] OR "aged, 80 and over"[Title/Abstract] OR "older person*"[Title/Abstract] OR "centenarian*"[MeSH Terms] OR "centenarian*"[Title/Abstract] OR "sexagenarian*"[Title/Abstract] OR "septuagenarian*"[Title/Abstract] OR "octogenarian*"[Mesh Terms] OR "octogenarian*"[Title/Abstract] OR "nonagenarian*"[MeSH Terms] OR "nonagenarian*"[Title/Abstract])**	3,922,024	05:26:00

Figure S3: Specific search algorithm for Pubmed
